# COVID-19 Serosurvey of Frontline Healthcare Workers in Western Australia

**DOI:** 10.1007/s44197-022-00065-1

**Published:** 2022-09-21

**Authors:** Herbert Ludewick, Rebecca Hahn, Claire Italiano, Lynette Pereira, Daniel Fatovich, Jemma Saxton, Richard Hunt, Kwok M. Ho, Peter Boan, Warren Pavey

**Affiliations:** 1 Heart and Lung Research Institute of Western Australia Inc, Harry Perkins Institute of Medical Research, 5 Robin Warren Drive, Perth, WA Australia; 2 Department of Infectious Diseases, Royal Perth Hospital, Perth, WA Australia; 3 Department of Microbiology, PathWest Laboratory Medicine, Fiona Stanley Hospital, Perth, WA Australia; 4 Department of Emergency Medicine, Royal Perth Hospital, University of Western Australia, Perth, WA Australia; 5 Centre for Clinical Research in Emergency Medicine, Harry Perkins Institute of Medical Research, Perth, WA Australia; 6 Department of Anaesthesia, Fiona Stanley Hospital, Perth, WA Australia; 7 Department of Intensive Care, Royal Perth Hospital, Perth, WA Australia; 8 Department of Infectious Diseases, Fiona Stanley Hospital, Perth, WA Australia; 9 Department of Microbiology, PathWest Laboratory Medicine WA, Fiona Stanley Hospital, Perth, 11 Robin Warren Dve, Murdoch, WA 6150 Australia

**Keywords:** COVID-19, Healthcare worker, Infection prevention, Infection control, Personal protective equipment, Serology

## Abstract

We aimed to study COVID-19 infection in healthcare workers (HCWs) during the first wave in a setting of low community incidence prior to HCW vaccination. We performed a cross-sectional study of frontline HCWs in two tertiary hospitals in Western Australia with questionnaire and testing for SARS-CoV-2 IgG antibodies, using a screening assay followed by confirmatory assays for initial reactive results. 799 Frontline HCWs were enrolled in the study, working in the emergency department (*n* = 194, 24.2%), ICU (*n* = 176, 22.0%), respiratory ward (*n* = 20, 2.5%), COVID clinic (*n* = 37, 4.6%), and theatre (*n* = 222, 28%). 189 (23.6%) were doctors, 327 (41.0%) nurses, and 283 (35.4%) other. Contact with a known COVID-19-positive patient occurred at work for 337 (42.1%), and outside work for 10 (1.2%). Four were diagnosed with COVID-19 by PCR, acquired overseas in two cases and related to healthcare work in two cases (one acquired from a colleague and one possibly acquired from patient contact in the healthcare setting). Nine HCWs had reactive screening serology, and three had confirmed positive IgG (these three were PCR-positive cases). Infection control procedures in the setting of low community incidence were effective at preventing HCW acquisition of COVID-19 infection.

## Introduction

In some settings, the risk of acquiring COVID-19 is higher for healthcare workers (HCWs), particularly those involved in the direct care of infected patients [1]. In a high prevalence population, HCWs may also acquire COVID-19 at work due to non-patient contact, or while not at work [2]. The prevention of COVID-19 in a healthcare institution is mitigated by many factors including vaccination, physical distancing, handwashing, screening for infection, early diagnosis of infection, universal masking, quarantine, contact tracing, outbreak management, and building and ventilation design. Appropriate personal protective equipment (PPE) is also vitally important [3].

Droplets and aerosols are considered the predominant routes of SARS-CoV-2 transmission. Proximity as a key determinant of risk suggests that droplet is more common than aerosol transmission, and poor ventilation has been implicated in some instances of transmission over distance [4]. Surgical masks are designed to protect the wearer from large droplet and hazardous fluids, and restrict respiratory emissions from the wearer. They do not provide reliable protection from inhaling small particle aerosols. Respirators are designed and tested to protect against exposure to small particle aerosols as well as large droplets, are tight-fitting, and require individual fit testing and also seal testing with each use. Whether surgical masks provide adequate protection for routine patient interaction is debated [5]. Some guidelines suggest respirators for all interactions with COVID-19-positive patients [6], and others for situations such as prolonged contact, where physical distancing cannot be maintained, or where patients are screaming or shouting [7].

At the beginning of the pandemic which is the period of this study, routine PPE in Australia was surgical mask, eye protection, gown and gloves, donned before entering the single room of a patient suspected or diagnosed with COVID-19. N95/P2 respirators or powered air purifying respirators (PAPRs) in place of a surgical mask were suggested only for aerosol generating procedures (AGPs).

Western Australia experienced the first wave of COVID-19 infection March to May 2020 and has had sporadic cases related to travel until the Omicron wave in 2022. During the first wave, there was no significant community transmission, so HCW acquisition of COVID-19 would very likely have occurred at work. As there is a significant percentage of asymptomatic (33% in one review [8]) or mild illness, a serological survey offers a more sensitive evaluation of infection and therefore of the effectiveness of hospital infection control procedures. We hypothesized that asymptomatic COVID-19 infection might not be rare among frontline HCWs who were in clinical contact with either symptomatic or asymptomatic COVID-19 patients during their routine clinical work. In this study, we aimed to assess the seroprevalence of COVID-19 among a wide range of frontline HCWs before vaccination.

## Methods

### Participants

The study was advertised through email and posters in mid-May 2020 to frontline healthcare workers (Emergency Department, Intensive Care Unit, Respiratory Ward, Anaesthetics) at Fiona Stanley Hospital (FSH) and Royal Perth Hospital (RPH), two large tertiary hospitals in Western Australia. All who were interested in participation were enrolled in June (FSH) and August (RPH) 2020. Enrolled participants completed a standardised questionnaire, focussing on epidemiological risk in and out of the hospital environment, and diagnosis of COVID-19 by PCR (Supplementary Appendix). Serum was taken at one occasion for each participant for SARS-CoV-2 antibody testing in June (FSH) and August (RPH) 2020.

### SARS-CoV-2 Antibody Testing

The serology screening assay used to detect SARS-CoV-2 antibodies was previously described [9–11]. Briefly, plasmids used to generate recombinant proteins derived from SARS-CoV-2 spike protein were gifted from Dr. Florian Krammer (Icahn School of Medicine, Mount Sinai, New York, USA). Recombinant receptor-binding domain (RBD) and full spike proteins were produced in-house as previously described.

The two-stage serological enzyme-linked immunosorbent assay (ELISA) previously described was used to screen serum from study subjects [10, 11]. The serum samples were first screened for the presence of SARS CoV-2 IgG antibodies samples on plates coated with RBD antigen. Reactive serum to RBD with optical density at 490 nm (OD_490_**)** ≥ 0.15 was then subjected to a second ELISA with SARS CoV-2 full-length spike protein. Here, serum samples were serially diluted and added to a spike antigen-coated plate. Samples were considered reactive for SARS-CoV-2 antibodies with an OD_490_ signal three standard deviations higher than the negative control in at least two dilutions [10, 11].

Sera reactive by the screening study assay (reactive for antibodies to both RBD and full-length spike protein) was further tested at the public hospital pathology provider, PathWest Laboratory Medicine WA. Serum was tested with the Abbott Architect SARS-CoV-2 IgG qualitative assay (Abbott Diagnostics, Australia) according the manufacturer’s instructions, which tests for antibodies against nucleocapsid protein, and a positive result when sample/cut-off value is ≥ 1.4. Serum was also tested by Euroimmun SARS-CoV-2 IgG assay (Lüberk, Germany) according the manufacturer’s instructions, which tests for IgG antibodies to the S1 domain of the spike protein, and a positive result when the sample/cut-off value is ≥ 1.1.

Positive sera were those with reactive results on the screening study assay (for antibody to both RBD and full-length spike protein) and one of the supplemental assays (Abbott Architect or Euroimmun).

Positive control for RBD and full-length spike protein screening assays was pooled convalescent sera from PCR-positive individuals in Western Australia.

Negative control sera for the screening assays was intravenous immunoglobulin (CSL Behring, Australia) obtained from plasma of > 100 donors prior to December 2019.

Cross-reactivity was assessed for the RBD screening assay with pre-2020 serum samples from patients with serum drawn for creatinine concentration, cytomegalovirus (CMV) serology, or hepatitis C virus (HCV) serology.

## Results

During the first wave of infection in Western Australia from March to May 2020, there were 21 (eight admitted to ICU) COVID-19 cases admitted to FSH and 30 (four admitted to ICU) to RPH. For 39 patients (11 admitted to ICU), information about the duration of admission could be obtained. They were admitted for median seven (interquartile range [IQR] 3.5–19.5) days, and those patients admitted to ICU for median 14 (IQR 8–22.5) days.

Of the 799 HCWs enrolled (398 from FSH and 401 from RPH), 42.1% reported direct contact with a COVID-19-positive patient at work, and 1.2% reported contact with a COVID-19-positive person outside work. Twenty percent had at least one respiratory illness and 11.5% had travelled overseas since 1 February 2020. Four participants had been diagnosed with COVID-19 by PCR, two acquired through overseas travel and two associated with healthcare work (Table 1.). One healthcare-associated case (case 426) was acquired from a colleague who had returned from the UK early in the pandemic before it was listed as a risk country. This colleague developed symptoms within 24 h of contact with case 426 which was close but less than 15 min, prompting testing of case 426 which returned positive. The individuals had matching SARS-CoV-2 type by whole-genome sequencing. The second potential healthcare-associated case (case 739) was a nurse in an Emergency Department who had contact with a COVID-19-positive case in appropriate PPE, without AGPs. The nurse also performed COVID-19 testing at Perth airport. Whole-genome sequencing and epidemiological investigation were inconclusive about the origin of her infection, which could have been at the Emergency Department or the airport. There were no other PCR-positive cases in HCWs detected at either hospital during the study period.

**Table 1:** Demographics of healthcare workers enrolled in the study

	Number (%)
Age (median)	41
FSH	398 (49.8)
RPH	401 (50.2)
Ward	
ED	194 (24.2)
ICU	176 (22.0)
Respiratory	20 (2.5)
COVID testing clinic	37 (4.6)
Theatre	222 (28.0)
Other	150 (18.7)
Role	
Doctor	189 (23.6)
Nurse	327 (41.0)
Other	283 (35.4)
Contact with COVID-19 at work	337 (42.1)
Contact with COVID-19 outside work	10 (1.2)
SARS-CoV-2 PCR test	247 (30.9)
Respiratory illness	160 (20.0)
Overseas travel	92 (11.5)
Total	799 (100)

*FSH* Fiona Stanley Hospital, *RPH* Royal Perth Hospital, *ED* Emergency department, *ICU* Intensive-care unit

**Fig. 1:**
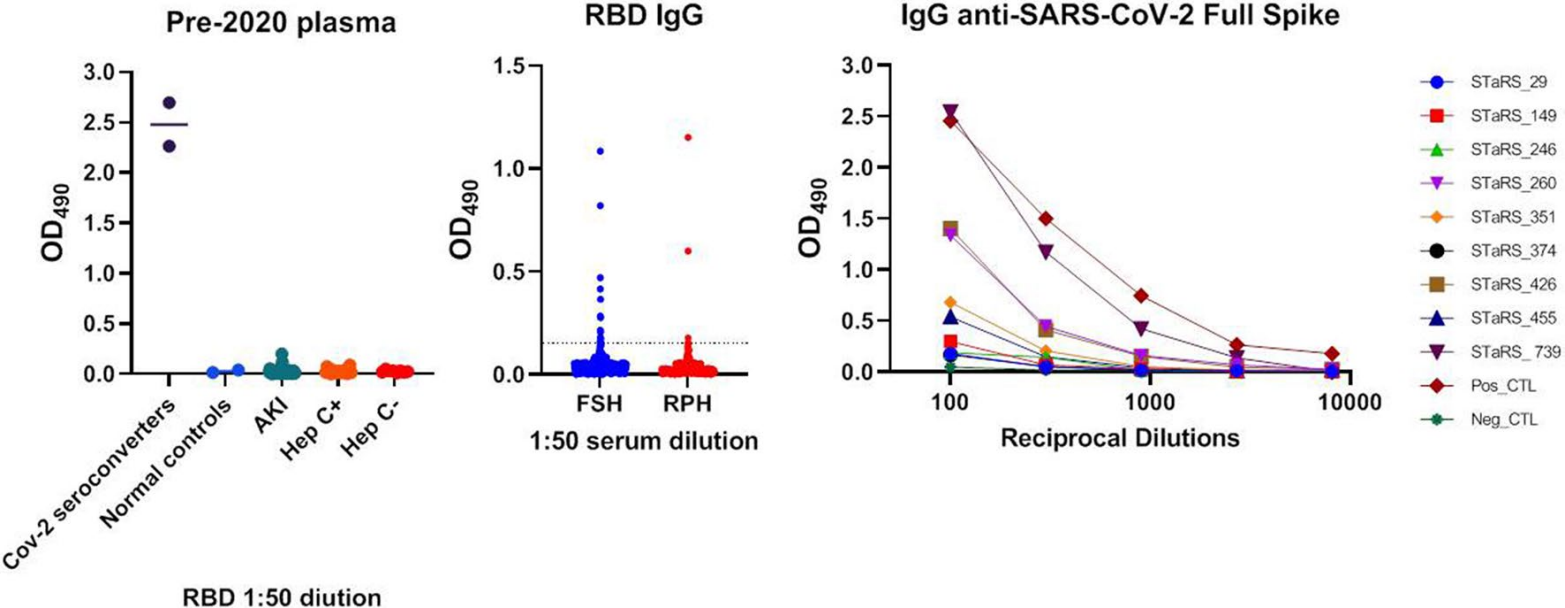
**A** Optical density at 490 nm (OD_490_) in the RBD IgG screening assay for positive, negative, and cross-reactivity controls. **B** OD_490_ for healthcare workers at Fiona Stanley Hospital and Royal Perth Hospital. **C** OD_490_ of serial dilutions for the Full Spike Protein screening IgG assay for the nine healthcare workers with reactive RBD IgG (OD_490_ ≥ 0.15)

There was no evidence of cross-reactivity for the RBD screening assay, and positive and negative controls gave predicted results on RBD and full-length spike protein screening assays (Fig. 1A and C).

Six participants at FSH and three participants at RPH had reactive tests for both RBD and full-length spike protein antibodies on the screening assay (Fig. 1B and C), of which three were positive by the Abbott Architect or Euroimmun assay (all three were PCR-positive). One PCR-positive participant (case number 149) tested reactive on the screening serological assays but negative on supplemental assays. For the PCR-positive cases, the time from positive PCR to the study serum testing was 84–148 days. See Table 2.

**Table 2:** Data of those healthcare workers with reactive serology on the screening serological Receptor-Binding Domain (RBD) assay

Case no	Site	PCR diagnosis	Acquisition	Contact with COVID-19	Overseas travel	RBD OD	Arch. S/CO	Euro S/CO
260	FSH	Yes	Overseas	Yes	Yes	1.09	1.76	2.2
149	FSH	Yes	Overseas	No	Yes	0.16	0.72	0.3
29	FSH	No	–	No	No	0.36	0.02	0.1
246	FSH	No	–	No	No	0.41	0.07	0.3
351	FSH	No	–	Yes	No	0.82	0.13	0.0
374	FSH	No	–	Yes	No	0.15	0.04	0.5
426	RPH	Yes	Occupational	Yes	No	0.60	5.72	4.2
739	RPH	Yes	Occupational	Yes	No	1.15	4.67	6.1
455	RPH	No	–	Yes	Yes	0.15	0.88	0.7

*OD* optical density. *Arch*. Abbott Architect SARS-CoV-2 qualitative IgG assay, *Euro*. Euroimmun SARS-CoV-2 IgG assay, *S/CO* sample:cut-off ratio, *FSH* Fiona Stanley Hospital, *RPH* Royal Perth Hospital

Architect is positive when S/CO ≥ 1.4 and Euroimmun when S/CO ≥ 1.1, with a confirmed serological positive if either test is positive

## Discussion

We performed a serosurvey in 799 frontline HCWs in Western Australia and found three participants with confirmed positive IgG antibodies to SARS-CoV-2, who all had confirmed infection by PCR. There was an additional participant with PCR-positive infection who did not have confirmed serology. Of the four individuals with COVID-19, two had acquired the infection overseas and two at work, one from a colleague and another possibly from patient contact. This reflects very little occupationally acquired infection and suggests the infection control procedures in place at that time were sufficient to prevent transmission from contact with infectious patients, including ICU patients who have been shown to have prolonged shedding at high viral loads [12]. Similar findings were published in Hong Kong where no nosocomial transmission occurred by day 72 of the pandemic with 130 cases diagnosed in the country. All cases had been admitted to hospital when N95 respirators were used for the care of suspected and confirmed cases [13]. Studies showing higher rates of HCW acquisition of COVID-19 have generally been in areas of significant community transmission, when infection control procedures become tested by more opportunities for transmission and there is significant risk of exposure at home and through contact with infected colleagues. One study showed similar seropositive rates for staff regardless of occupational exposure risk and a higher seropositivity rate in those exposed in the community, suggesting that the community was the primary place of acquisition [14]. On the contrary, a study of HCW infection at Royal Melbourne Hospital showed higher rates of infection in staff from wards managing COVID-19 patients, and in staff with more patient contact (e.g., nurses), suggesting acquisition of infection at work. PPE breach was thought to be rare. Old ventilation systems, use of multi-bed rooms for infected patients, and the density of infected patients were contributors to infection of HCW in spite of PPE [15]. Findings from a London hospital show that patients are also at risk of acquiring COVID-19 in a high prevalence setting, with 66 of 435 inpatients with COVID-19 deemed to have acquired the infection in hospital [16]. Comparatively, FSH and RPH only had a small number of inpatients with COVID-19.

With low density of infection in the hospitals, surgical mask, eye protection, and contact precautions in the absence of AGPs protected HCWs from acquiring infection during the first wave of infection, apart from a possible single instance. Of course, instances of PPE failure have been documented in conditions such as high patient viral load, crowding, or poor ventilation. An example is infection from an undiagnosed COVID-19 case on steroids and nebulised medications to a speech pathologist during video fluoroscopy and to a radiology technician who both wore surgical masks, eye protection, and gloves [17]. In another instance, six HCWs acquired infection from an infected child and her mother in the absence of AGPs and despite PPE including surgical masks. Aerosol transmission was thought be likely due to the distance of some HCWs from the index patients [18]. It should be noted that our study was performed at a time when variants of concern were not circulating. These have been shown to be more transmissible [19] and are a factor in proposing N95/P2 respiratory use for standard patient care in the absence of AGPs.

There are limitations to this study. Enrolment was voluntary and some participants did not have direct patient contact (e.g., ward clerks) as noted in the results. Additionally, there were not a high number of patients admitted to either hospital, nor to the ICU. Knowing the potential low positive predictive value of assays in a low pre-test probability environment, we utilised a tiered approach to serological testing to improve specificity and predictive value, which is suggested by the Public Health Laboratory Network of Australia. We acknowledge that finding very infrequent occupational acquisition of COVID-19 may not be generalizable to variants of concern with increased transmissibility.

## Conclusions

We demonstrated very infrequent HCW occupational acquisition of COVID-19 during the first wave of infection in Western Australia, suggesting that the infection control procedures in place at that time were effective at preventing infection with the initial strains.

## Data Availability

Readers can contact the corresponding author for access to data.

## Abbreviations

AGP: Aerosol generating procedure
CMV: Cytomegalovirus
ELISA: Enzyme-linked immunosorbent assay
FSH: Fiona Stanley Hospital
HCV: Hepatitis C virus
HCW: Healthcare worker
ICU: Intensive-care unit
IQR: Interquartile range
OD_490_: Optical density at 490 nm
PAPR: Powered air purifying respirators
PCR: Polymerase chain reaction
PHLN: Public Health Laboratories Network
PPE: Personal protective equipment
RBD: Receptor-binding domain
RPH: Royal Perth Hospital
SN: Sensitivity
SP: Specificity
